# An exploration of causal relationships between nine neurological diseases and the risk of breast cancer: a Mendelian randomization study

**DOI:** 10.18632/aging.205745

**Published:** 2024-04-24

**Authors:** Fei Ren, Chenxuan Yang, Kexin Feng, Qingyao Shang, Jiaxiang Liu, Xiyu Kang, Xin Wang, Xiang Wang

**Affiliations:** 1 Department of Breast Surgical Oncology, National Cancer Center/National Clinical Research Center for Cancer/Cancer Hospital, Chinese Academy of Medical Sciences and Peking Union Medical College, Beijing 100021, China

**Keywords:** Mendelian randomization, causal relationship, Alzheimer’s disease, multiple sclerosis, breast cancer

## Abstract

Background: Some preceding researches have observed that certain neurological disorders, such as Alzheimer’s disease and multiple sclerosis, may affect breast cancer risk. However, whether there are causal relationships between these neurological conditions and breast cancer is inconclusive. This study was designed to explore whether neurological disorders affected the risks of breast cancer overall and of the two subtypes (ER+ and ER-).

Methods: In the course of this study, genome-wide association study (GWAS) data for nine neurological diseases (Alzheimer’s disease, multiple sclerosis, Parkinson’s disease, myasthenia gravis, generalized epilepsy, intracerebral haemorrhage, cerebral atherosclerosis, brain glioblastoma, and benign meningeal tumour) were collected from the Complex Trait Genetics lab and the MRC Integrative Epidemiology Unit, and single-nucleotide polymorphisms (SNPs) extensively associated with these neurological ailments had been recognized as instrumental variables (IVs). GWAS data on breast cancer were collected from the Breast Cancer Association Consortium (BCAC). Two-sample Mendelian randomization (MR) analyses as well as multivariable MR analyses were performed to determine whether these SNPs contributed to breast cancer risk. Additionally, the accuracy of the results was evaluated using the false discovery rate (FDR) multiple correction method. Both heterogeneity and pleiotropy were evaluated by analyzing sensitivities.

Results: According to the results of two-sample MR analyses, Alzheimer’s disease significantly reduced the risks of overall (OR 0.925, 95% CI [0.871−0.982], *P* = 0.011) and ER+ (OR 0.912, 95% CI [0.853−0.975], *P* = 0.007) breast cancer, but there was a negative result in ER- breast cancer. However, after multiple FDR corrections, the effect of Alzheimer’s disease on overall breast cancer was not statistically significant. In contrast, multiple sclerosis significantly increased ER+ breast cancer risk (OR 1.007, 95% CI [1.003-1.011], *P* = 0.001). In addition, the multivariable MR analyses showed that Alzheimer’s disease significantly reduced the risk of ER+ breast cancer (IVW: OR 0.929, 95% CI [0.864-0.999], *P*=0.047; MR-Egger: OR 0.916, 95% CI [0.846-0.992], *P*=0.031); however, multiple sclerosis significantly increased the risk of ER+ breast cancer (IVW: OR 1.008, 95% CI [1.003-1.012], *P*=4.35×10^-4^; MR-Egger: OR 1.008, 95% CI [1.003-1.012], *P*=5.96×10^-4^). There were no significant associations between the remainder of the neurological diseases and breast cancer.

Conclusions: This study found the trends towards a decreased risk of ER+ breast cancer in patients with Alzheimer’s disease and an increased risk in patients with multiple sclerosis. However, due to the limitations of Mendelian randomization, we cannot determine whether there are definite causal relationships between neurological diseases and breast cancer risk. For conclusive evidences, more prospective randomized controlled trials will be needed in the future.

## INTRODUCTION

As the most common form and the primary cause of death for women with cancers, breast cancer has varying degrees of impacts on women’s quality of life and survival [1, 2]. Some correlations have been found between cancers and neurological diseases, like Alzheimer’s disease and multiple sclerosis [3–5]. Based on previous researches, Alzheimer’s disease was negatively associated with cancers. As compared to the control group, Alzheimer’s patients were 42-50% less likely to develop cancers, and cancer patients were also less likely to develop Alzheimer’s [6–10]. Another study indicated that breast cancer patients had a lower risk of having previously had Alzheimer’s [11]. Studies of large populations have shown an increased cancer rate among people with multiple sclerosis [12–14], whereas other studies did not pinpoint a clear connection [15, 16]. Experimental designs or observational studies in the past may have been limited or confounded by some factors, resulting in different conclusions. There are, therefore, no clear causal relationships between certain neurological diseases and cancer risks.

It is a promising epidemiological method for determining exposure-outcome relationships through Mendelian randomization (MR) [5, 17, 18]. According to Mendel’s Second Law, random classifications of alleles during the process of gametic formation can lead to random allocations of exposures associated with an allele or group of alleles, which are usually independent of environmental risk factors and precede risk factors and disease progression [19, 20]. In MR, genetic variables act as instrumental variables (IVs) [21–23]. Like randomized controlled trials, MR makes use of single-nucleotide polymorphisms (SNPs) to randomly divide individuals into two companies described via genotype, and it assumes that genotype distribution is a random action in the course of meiosis, making MR less affected by possible confounders and reverse causalities [24, 25]. In MR analysis, three assumptions must be taken into account: (1) genetic variants that are considered as IVs should be strongly correlated with the exposure; (2) it is imperative that no confounding factors are linked to the genetic variants used; and (3) the selected genetic variants should affect the outcome only through the exposure, not via other means [26–28].

Based on genome-wide association study (GWAS) statistics, we systematically investigated the causal relationships between nine neurological diseases and breast cancer risk using MR analyses. Our findings may offer some insights into breast cancer screening and treatment strategies.

## MATERIALS AND METHODS

### GWAS data for neurological diseases

Nine neurological disorders were selected for this study, including Alzheimer’s disease, multiple sclerosis, Parkinson’s disease, myasthenia gravis, generalized epilepsy, intracerebral haemorrhage, cerebral atherosclerosis, brain glioblastoma, and benign meningeal tumour. We retrieved the GWAS data for Alzheimer’s disease from the Complex Trait Genetics lab (https://ctg.cncr.nl/software/summary_statistics); GWAS data for multiple sclerosis came from the International Multiple Sclerosis Genetics Consortium; GWAS data for Parkinson’s disease came from the International Parkinson’s Disease Genomics Consortium; and for the remaining neurological diseases, the data were from the FinnGen consortium, which can be publicly accessed from the MRC Integrative Epidemiology Unit (https://gwas.mrcieu.ac.uk/). The GWAS data for all neurological disorders came from a population of European descent. Supplementary Table 1 describes the information related to GWAS data for these nine neurological disorders.

### GWAS data for breast cancer

GWAS data on overall breast cancer and its subtypes (ER+ and ER-) for Europeans, including 61282 breast cancer patients (38197 ER+ cases and 9655 ER- cases) and 45494 controls, were obtained from the Breast Cancer Association Consortium (BCAC) and were publicly available on the website https://gwas.mrcieu.ac.uk/. Supplementary Table 2 describes the detailed information on the GWAS data for breast cancer. A detailed description of diagnostic criteria, demographic characteristics, and quality control can be found in the original GWAS [29].

### Instrumental variable selection

For Alzheimer’s disease, multiple sclerosis, and Parkinson’s disease, we chose single-nucleotide polymorphisms (SNPs) that independently affected these neurological disorders at a genome-wide significance level (P<5×10^-8^) and were not in linkage disequilibrium (LD, r^2^ < 0.1) for the Mendelian randomization analyses. However, as only a few SNPs reached genome-wide significance for the remaining neurological diseases, we relaxed the association threshold, with P<5×10^-6^ and LD r^2^ < 0.001. Earlier studies have used this method [30–32]. We calculated the phenotypic variance explained through every instrument with R^2^: R^2^=[2×EAF×(1-EAF)×(β)^2^]/[(2×EAF×(1-EAF)×(β)^2^)+(2×EAF×(1-EAF)×N×se(β)^2^)], where EAF was the effect allele frequency, β was the estimated genetic effect on neurological diseases, N was the sample size and se (β) was the standard error of the genetic effect. We additionally calculated the F-statistic to examine the statistical strength of every instrumental variable by the following formula: F=[R^2^×(N-k-1)]/[(1-R^2^)×k], and k was the number of instrumental variables [32–34]. F>10 indicated that the instrumental variables were robust and could be used for MR analyses [19, 35, 36]. Next, we extracted SNPs for neurological diseases from the breast cancer data and eliminated the ones associated with outcomes. A coordination process was then carried out to align SNP alleles between exposures and outcomes, and we discarded palindromic SNPs with medium effective allelic frequencies or SNPs with incompatible alleles. Then, we screened each SNP strongly associated with neurological diseases in the Phenoscanner V2 website (http://www.phenoscanner.medschl.cam.ac.uk/) to explore and eliminate those SNPs related to common confounding factors, including age of menarche [37], alcohol intake frequency [38], oestrogen [39] and mammographic density [40]. MR analyses were only conducted on exposures containing more than 3 SNPs.

### Statistical analyses

In the two-sample MR analysis, odds ratios (ORs) and 95% confidence intervals (CIs) were calculated by the inverse variance weighting (IVW) method [19, 31]. MR analyses commonly used the IVW method to pool all Wald ratios for every SNP [41]. The IVW assumed all genetic variants were valid, making it the most effective MR estimation method, while it was shown to be susceptible to pleiotropic bias. Causal links between neurological diseases and breast cancer risk were examined using IVW as the primary method of evaluation in our study. Furthermore, MR-Egger method was applied along with weighted median method. For the purpose of assessing horizontal pleiotropy, the MR-Egger regression approach was employed if the intercept value deviated from zero [42]. To make the results more reliable, false discovery rate (FDR) multiple testing method was applied to obtain adjusted *P*-values. *P*<0.05 represented statistical significance.

For detecting the heterogeneity, we used the Cochran Q test, which confirmed that differences among effect sizes in selected genetic variants were not due to sampling errors, but to actual differences among SNPs [1, 43, 44]. *P*<0.05 indicated that heterogeneity was present. Horizontal pleiotropy value was assessed on the basis of Egger intercepts [35, 42]. Furthermore, we carried out leave-one-out (LOO) analyses in order to identify the high interference points that drove the pooled IVW estimates.

As Alzheimer’s disease and multiple sclerosis are genetically linked, there may have been false-positive results in the two-sample MR analyses. We then conducted multivariable IVW and multivariable MR-Egger analyses so that we could assess the causal connections between these two diseases and breast cancer. Using the “MendelianRandomization”, “TwoSampleMR”, “data.table”, “VariantAnnotation” packages of R version 4.2.3, statistical analyses were performed.

## RESULTS

### Correlations between neurological diseases and overall breast cancer risk

**Figure 1:**
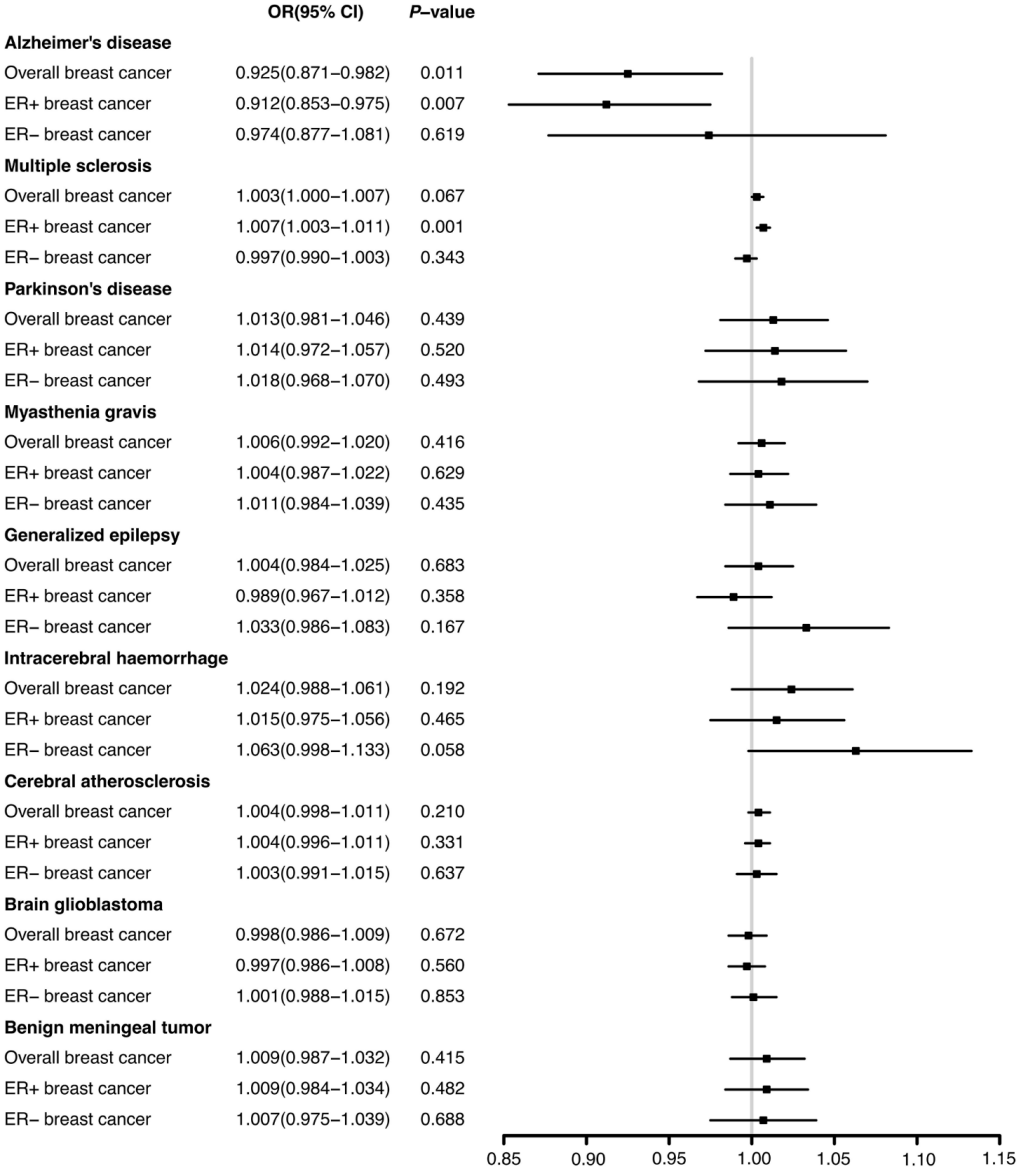
The effects of nine neurological diseases on the risks of overall, ER+ and ER- breast cancer from IVW method.

**Figure 2:**
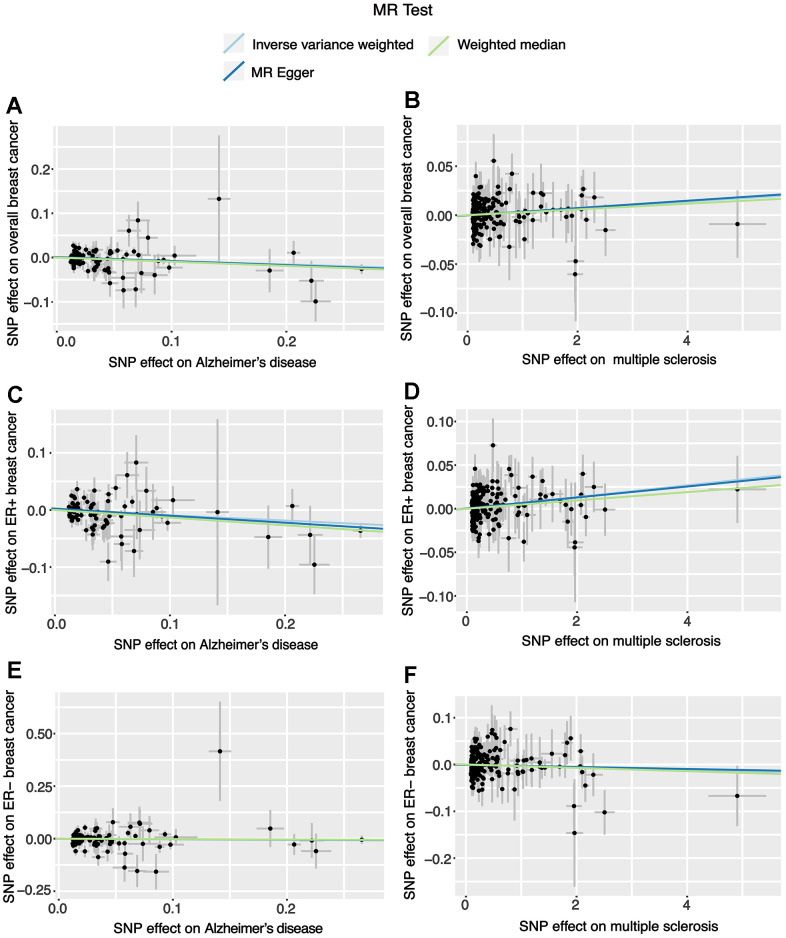
**Scatter plots for the effects of Alzheimer’s disease and multiple sclerosis on breast cancer risk.** (**A**) The effect of Alzheimer’s disease on overall breast cancer; (**B**) The effect of multiple sclerosis on overall breast cancer; (**C**) The effect of Alzheimer’s disease on ER+ breast cancer; (**D**) The effect of multiple sclerosis on ER+ breast cancer; (**E**) The effect of Alzheimer’s disease on ER- breast cancer; (**F**) The effect of multiple sclerosis on ER- breast cancer.

Through the two-sample MR analysis using IVW method, we found that Alzheimer’s disease significantly reduced the overall breast cancer risk (OR 0.925, 95% CI [0.871−0.982], *P* = 0.011) (Figure 1). According to the scatterplot, we were able to see the causal estimates that were generated from each instrumental variable (Figure 2). A similar conclusion was reached with the MR-Egger and weighted median methods (Table 1). However, after FDR multiple corrections, the adjusted *P*-values showed that Alzheimer’s disease was not associated with a significantly lower breast cancer risk (Table 2). Neither heterogeneity nor pleiotropy was observed (Table 1). The LOO analysis revealed that none of the instrumental variables significantly altered the degree of causality between Alzheimer’s disease and overall breast cancer risk (Supplementary Figure 1). For multiple sclerosis, Parkinson’s disease, myasthenia gravis, generalized epilepsy, intracerebral haemorrhage, cerebral atherosclerosis, brain glioblastoma, and benign meningeal tumours, there was no evidence that they could significantly affect the overall breast cancer risk (Table 1 and Figure 1).

**Table 1:** The MR analyses of neurological diseases and overall breast cancer risk from MR Egger and weighted median methods.

**Neurological diseases**	**Used SNPs**	**MR Egger**			**Weighted median**		***P*_heterogeneity_**	***P*_pleiotropy_**
		**OR(95% CI)**	***P*-value**		**OR(95% CI)**	***P*-value**		
Alzheimer’s disease	86	0.913(0.843-0.989)	0.028		0.909(0.842-0.982)	0.015	0.070	0.626
Multiple sclerosis	192	1.004(0.999-1.009)	0.121		1.003(0.998-1.008)	0.272	0.028	0.848
Parkinson’s disease	24	1.038(0.959-1.125)	0.366		1.026(0.984-1.070)	0.225	0.119	0.509
Myasthenia gravis	8	1.001(0.980-1.023)	0.909		1.004(0.988-1.020)	0.639	0.317	0.584
Generalized epilepsy	12	1.019(0.990-1.048)	0.237		1.005(0.980-1.031)	0.705	0.850	0.197
Intracerebral haemorrhage	5	1.022(0.964-1.083)	0.518		1.027(0.982-1.073)	0.245	0.457	0.938
Cerebral atherosclerosis	7	1.005(0.997-1.013)	0.290		1.004(0.996-1.012)	0.297	0.871	0.777
Brain glioblastoma	8	0.993(0.974-1.012)	0.495		0.997(0.988-1.007)	0.616	0.016	0.567
Benign meningeal tumor	12	0.992(0.964-1.022)	0.629		1.002(0.978-1.027)	0.871	0.112	0.153

### Correlations between neurological disorders and the risk of ER+ breast cancer

There was a significant reduction in the risk of breast cancer with ER+ in patients with Alzheimer’s disease (OR 0.912, 95% CI [0.853−0.975], *P* = 0.007) according to IVW method from the two-sample MR analysis. In contrast, multiple sclerosis significantly increased ER+ breast cancer risk (OR 1.007, 95% CI [1.003-1.011], *P* = 0.001) (Figure 1). The correlation values estimated using the MR-Egger and weighted median approaches were generally in agreement with those computed by IVW (Supplementary Table 3). The results after FDR multiple corrections were consistent with those described above (Table 2). No significant heterogeneity was observed. Moreover, according to the MR-Egger test, no significant pleiotropic effects were observed among the genetic instrumental variables (Supplementary Table 3). The LOO figures revealed no significant influences of instrumental variables on the causal correlations between these two neurological ailments and ER+ breast cancer (Supplementary Figures 2, 3). Additionally, the remaining seven neurological diseases had no significant impacts on ER+ breast cancer risk (Figure 1 and Supplementary Table 3).

**Table 2:** The adjusted *P*-values after the multiple corrections using the FDR method.

**Neurological diseases**	**Overall breast cancer**	**ER+ breast cancer**
Alzheimer’s disease	0.099	0.032
Multiple sclerosis	0.302	0.009
Parkinson’s disease	0.564	0.629
Myasthenia gravis	0.564	0.629
Generalized epilepsy	0.683	0.629
Intracerebral haemorrhage	0.473	0.629
Cerebral atherosclerosis	0.473	0.629
Brain glioblastoma	0.683	0.629
Benign meningeal tumor	0.564	0.629

The adjusted *P*-values were obtained based on the *P*-values from IVW method.

**Figure 3:**
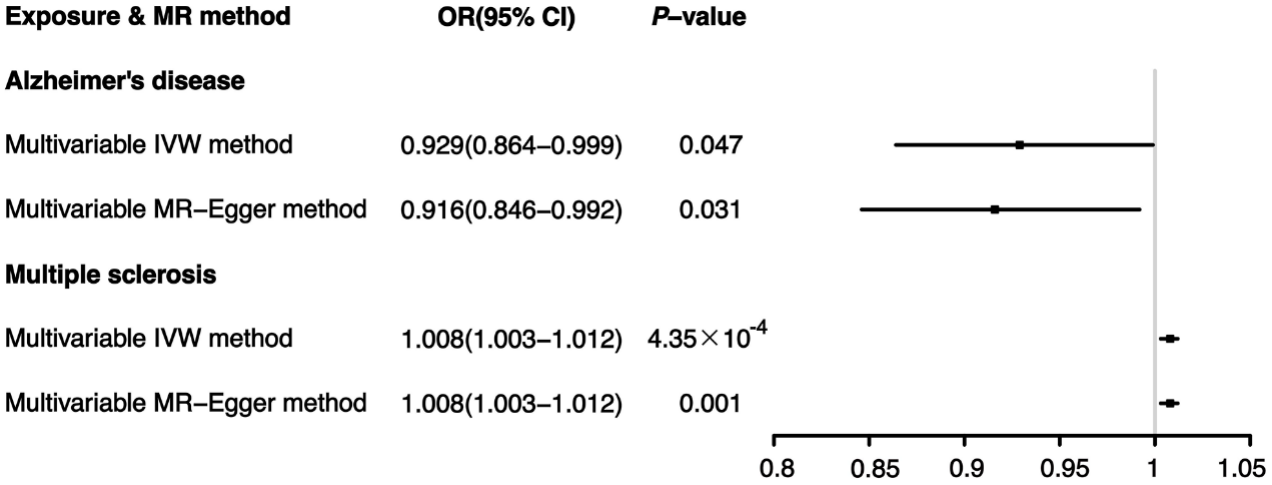
Effects of Alzheimer’s disease and multiple sclerosis on ER+ breast cancer: results from the multivariable MR analysis.

Then, a multivariable MR analysis for Alzheimer’s disease and multiple sclerosis was performed, with the outcome of ER+ breast cancer. Both the multivariable IVW method and the multivariable MR-Egger method indicated that Alzheimer’s disease significantly reduced the risk of ER+ breast cancer (IVW: OR 0.929, 95% CI [0.864-0.999], *P*=0.047; MR-Egger: OR 0.916, 95% CI [0.846-0.992], *P*=0.031). In contrast, breast cancer with ER+ was significantly more likely to occur in individuals with multiple sclerosis (IVW: OR 1.008, 95% CI [1.003-1.012], *P*=4.35×10^-4;^ MR-Egger: OR 1.008, 95% CI [1.003-1.012], *P*=5.96×10^-4^), according to the multivariable MR analysis (Figure 3).

### Correlations between neurological disorders and the risk of ER- breast cancer

According to the results of two-sample MR analyses, none of the nine neurological disorders significantly affected the risk of ER- breast cancer (Figure 1). The results obtained from the MR-Egger and weighted median resembled those obtained from IVW. Heterogeneity and pleiotropy were not shown (Supplementary Table 4).

## DISCUSSION

In this research, the causal connections between nine neurological disorders and breast cancer were investigated using two-sample MR and multivariable MR analyses. We found that Alzheimer’s disease significantly reduced overall and ER+ breast cancer risks. Although the effect of Alzheimer’s disease on overall breast cancer after FDR multiple corrections was not statistically significant, it still suggested a tendency for Alzheimer’s disease to reduce breast cancer risk, in line with previous findings [7, 11]. In addition, this study also demonstrated that multiple sclerosis can significantly increase ER+ breast cancer risk, which was also concluded in previous researches [13]. The other seven neurological disorders did not appear to be associated with the risk of breast cancer.

This study provided some evidences that Alzheimer’s disease and breast cancer were genetically linked. The pathophysiological mechanisms of these two diseases have been extensively researched but have not been clearly defined. Alzheimer’s disease and cancers are negatively correlated, which indicates that one disease may prevent the other. An Alzheimer’s patient’s cancer risk was 61% lower than that of a control participant [6]. Other studies have also demonstrated that Alzheimer’s patients were less likely to develop cancers [8–11]. Conversely, some comprehensive longitudinal studies with large numbers of participants also concluded that cancers might reduce Alzheimer’s risk [7, 45, 46].

Cancer and neurodegeneration are viewed as having opposite mechanisms: one involves a resistance to cell death, while the other involves premature cell death [47, 48]. The pathophysiology of Alzheimer’s disease plays a role in apoptosis, synaptic loss and neuronal dysfunction. The growth of cancer is uncontrolled and excessive, in contrast [49]. Both diseases share a few common risk factors, with aging being the most significant one. The key steps in its pathophysiology are dysregulation of the cell cycle and inflammation. Both diseases are characterized by mechanisms that regulate cell survival. Immune function and development can be adversely affected by aging. Metabolic disorders and reprogramming associated with aging may contribute to neurodegeneration and cancer development. Both ailments are related to pathways and genes concerned in bioenergetics, inflammation, DNA harm and repair, oxidative stress and unusual cell cycle activation [47, 50]. There are several other factors that contribute to both conditions, such as obesity, diabetes, physical inactivity, smoking and family history. Furthermore, Alzheimer’s disease and cancer also share some signalling pathways. Among the cyclins, p53 is a particularly important protein. Human cancers, such as breast cancer, frequently display dysfunctional p53 activity [51, 52]. Studies have indicated that conformationally altered p53 had novel transcriptional features, and this change was involved in cancer development by affecting genes that regulated transcriptional regulators responsible for encoding carcinogenic activities. The p53 gene has also been shown to be crucial in neurodegenerative diseases such as Alzheimer’s. In Alzheimer’s disease, the p53 controls various neuropathologic processes, such as lethal cell cycle reentry, immoderate DNA damages, and abnormal cell deaths. The Wnt signalling pathway is another related pathway. Researchers have found that suppressing Wnt signaling increased susceptibility to neuronal death while preventing cancer growth. Furthermore, upregulation of the Wnt pathway accelerated tumour development while also protecting against neurodegeneration [53, 54].

This study also revealed a link at the genetic level between multiple sclerosis and breast cancer. Although no statistical significance was observed in the MR analysis between multiple sclerosis and overall breast cancer, there was still a tendency for multiple sclerosis to increase ER+ breast cancer risk. In 2015, a systematic review found that cancers of the cervix, breast and digestive system had high incidences in multiple sclerosis patients [55]. In Sweden, women with multiple sclerosis aged 65 years and older were more likely to develop breast cancer [56, 57]. Numerous studies have found that cases and deaths from bladder cancer are higher in multiple sclerosis patients than in matched patients [55, 58]. Multiple sclerosis also appeared to be associated with an increased incidence of cancer in accordance with other previous studies [13, 14, 59]. The links between multiple sclerosis and cancers are complex, and there are several factors that may affect the morbidities of cancers. A person with multiple sclerosis is much more likely to smoke, be inactive, and be obese, which are all associated with a higher cancer risk [60–62]. Chronic immunosuppression that is secondary to the use of disease-modifying therapy (DMT) may also increase the risk of cancer [63–67]. Several studies found that the balance between inflammatory and regulatory T cells was disrupted in patients with multiple sclerosis, which correlated with the disease activity [68, 69]. It has been suggested that excessive inflammation of Th17 cells or excessive immunosuppression induced by Treg cells might cause cancers [70]. More evidence will be obtained by randomized controlled trials.

According to our research, Parkinson’s disease and breast cancer were not significantly related, which was in agreement with previous studies [71, 72]. Other studies, however, have linked Parkinson’s disease with a few types of cancers, such as lung cancer and pancreatic cancer [73–75]. It is possible, in the absence of causal effects, for apparent associations to be explained by confounding factors, genetic predispositions, biological pathways, or the biases identified during the evaluation processes [76, 77]. A definitive link between Parkinson’s disease and breast cancer should also be demonstrated by prospective trials. Moreover, the study did not find significant associations between several other neurological diseases and breast cancer. Previous studies demonstrated that elderly myasthenia gravis patients with a longer course of disease had higher risks of developing extrathyroidal malignancies [78]. We may have reached different conclusions due to the small sample sizes in the GWAS data. In addition, it is possible that previous results were biased or confounded by various factors. There are few studies on the relationships between other neurological diseases and cancers, and more accurate evidence needs to come from randomized controlled trials with large sample sizes.

This study has several obvious advantages. Firstly, this is the first time that causal relationships between nine neurological diseases and breast cancer have been assessed using two-sample MR and multivariable MR methods. In addition, MR analysis has the advantage of making public data more accessible, so research time and expenses can be reduced. Furthermore, the MR design minimizes reverse causality and residual confounding. In our study, multiple methods have been used to verify that MR assumptions were not violated in order to ensure that MR estimates were accurate. Different MR models showed similar directions and amplitudes, confirming the robustness. Despite this, our study has undeniable limitations. First, the GWAS data of breast cancer in this study came from female samples, so that we lack evidences on whether neurological diseases have effects on the risks of male breast cancer patients. Second, we didn’t explore the effects of breast cancer on neurological diseases in reverse, future researches can focus on this topic. Second, the study used European population data, and further explorations of the effects of neurological disorders on breast cancer risk in other populations will be needed in the future. Third, the sample size for each neurological disease in this study was different, resulting in different standards for the screening of SNPs required for our study. In the future, more data with large sample sizes will be needed for research. Fourth, although we tried to minimize the effects of pleiotropy in this study, it is impossible to completely eliminate pleiotropy in complex biological systems. The MR approach used in this study can only provide trends for the associations between neurological diseases and breast cancer risk, but cannot definitively confirm causal relationships. Therefore, more randomized controlled trials with large-sample data are needed to draw more accurate conclusions.

Overall, based on the results of this comprehensive MR study, Alzheimer’s disease tends to be negatively correlated with ER+ breast cancer, while multiple sclerosis has a trend towards a positive association, which can help the prevention of breast cancer in clinical practice. However, this study can only obtain trends rather than clear causal relationships between them on account of the limitations of MR analyses. In the future, the accurate results will be demonstrated with more prospective experimental designs, such as randomized controlled trials and cohort studies.

## Supplementary Material

Supplementary Figures

Supplementary Tables

## References

[1] Papadimitriou N, Dimou N, Gill D, Tzoulaki I, Murphy N, Riboli E, Lewis SJ, Martin RM, Gunter MJ, Tsilidis KK. Genetically predicted circulating concentrations of micronutrients and risk of breast cancer: A Mendelian randomization study. Int J Cancer. 2021; 148:646–53. 10.1002/ijc.33246 32761610 PMC8268064

[2] Johnson KE, Siewert KM, Klarin D, Damrauer SM, Chang KM, Tsao PS, Assimes TL, Maxwell KN, Voight BF, and VA Million Veteran Program. The relationship between circulating lipids and breast cancer risk: A Mendelian randomization study. PLoS Med. 2020; 17:e1003302. 10.1371/journal.pmed.1003302 32915777 PMC7485834

[3] Dong Z, Xu M, Sun X, Wang X. Mendelian randomization and transcriptomic analysis reveal an inverse causal relationship between Alzheimer’s disease and cancer. J Transl Med. 2023; 21:527. 10.1186/s12967-023-04357-3 37542274 PMC10403895

[4] Schwartz L, Peres S, Jolicoeur M, da Veiga Moreira J. Cancer and Alzheimer’s disease: intracellular pH scales the metabolic disorders. Biogerontology. 2020; 21:683–94. 10.1007/s10522-020-09888-6 32617766

[5] Melamed E, Lee MW. Multiple Sclerosis and Cancer: The Ying-Yang Effect of Disease Modifying Therapies. Front Immunol. 2020; 10:2954. 10.3389/fimmu.2019.02954 31998289 PMC6965059

[6] Zabłocka A, Kazana W, Sochocka M, Stańczykiewicz B, Janusz M, Leszek J, Orzechowska B. Inverse Correlation Between Alzheimer’s Disease and Cancer: Short Overview. Mol Neurobiol. 2021; 58:6335–49. 10.1007/s12035-021-02544-1 34523079 PMC8639554

[7] Driver JA. Inverse association between cancer and neurodegenerative disease: review of the epidemiologic and biological evidence. Biogerontology. 2014; 15:547–57. 10.1007/s10522-014-9523-2 25113739

[8] Li JM, Liu C, Hu X, Cai Y, Ma C, Luo XG, Yan XX. Inverse correlation between Alzheimer’s disease and cancer: implication for a strong impact of regenerative propensity on neurodegeneration? BMC Neurol. 2014; 14:211. 10.1186/s12883-014-0211-2 25394409 PMC4232711

[9] Ospina-Romero M, Glymour MM, Hayes-Larson E, Mayeda ER, Graff RE, Brenowitz WD, Ackley SF, Witte JS, Kobayashi LC. Association Between Alzheimer Disease and Cancer With Evaluation of Study Biases: A Systematic Review and Meta-analysis. JAMA Netw Open. 2020; 3:e2025515. 10.1001/jamanetworkopen.2020.25515 33185677 PMC7666424

[10] Sánchez-Valle J, Tejero H, Ibáñez K, Portero JL, Krallinger M, Al-Shahrour F, Tabarés-Seisdedos R, Baudot A, Valencia A. A molecular hypothesis to explain direct and inverse co-morbidities between Alzheimer’s Disease, Glioblastoma and Lung cancer. Sci Rep. 2017; 7:4474. 10.1038/s41598-017-04400-6 28667284 PMC5493619

[11] Freedman DM, Wu J, Chen H, Kuncl RW, Enewold LR, Engels EA, Freedman ND, Pfeiffer RM. Associations between cancer and Alzheimer’s disease in a U.S. Medicare population. Cancer Med. 2016; 5:2965–76. 10.1002/cam4.850 27628596 PMC5083750

[12] Ge F, Huo Z, Li C, Wang R, Wang R, Liu Y, Chen J, Lu Y, Wen Y, Jiang Y, Peng H, Wu X, Liang H, et al. Lung cancer risk in patients with multiple sclerosis: a Mendelian randomization analysis. Mult Scler Relat Disord. 2021; 51:102927. 10.1016/j.msard.2021.102927 33812221

[13] Hongell K, Kurki S, Sumelahti ML, Soilu-Hänninen M. Risk of cancer among Finnish multiple sclerosis patients. Mult Scler Relat Disord. 2019; 35:221–7. 10.1016/j.msard.2019.08.005 31404761

[14] Sun LM, Lin CL, Chung CJ, Liang JA, Sung FC, Kao CH. Increased breast cancer risk for patients with multiple sclerosis: a nationwide population-based cohort study. Eur J Neurol. 2014; 21:238–44. 10.1111/ene.12267 24053223

[15] Bahmanyar S, Montgomery SM, Hillert J, Ekbom A, Olsson T. Cancer risk among patients with multiple sclerosis and their parents. Neurology. 2009; 72:1170–7. 10.1212/01.wnl.0000345366.10455.62 19332695

[16] Nielsen NM, Rostgaard K, Rasmussen S, Koch-Henriksen N, Storm HH, Melbye M, Hjalgrim H. Cancer risk among patients with multiple sclerosis: a population-based register study. Int J Cancer. 2006; 118:979–84. 10.1002/ijc.21437 16152598

[17] Lawlor DA, Harbord RM, Sterne JA, Timpson N, Davey Smith G. Mendelian randomization: using genes as instruments for making causal inferences in epidemiology. Stat Med. 2008; 27:1133–63. 10.1002/sim.3034 17886233

[18] Luo S, Li W, Li Q, Zhang M, Wang X, Wu S, Li Y. Causal effects of gut microbiota on the risk of periodontitis: a two-sample Mendelian randomization study. Front Cell Infect Microbiol. 2023; 13:1160993. 10.3389/fcimb.2023.1160993 37305424 PMC10248501

[19] Burgess S, Butterworth A, Thompson SG. Mendelian randomization analysis with multiple genetic variants using summarized data. Genet Epidemiol. 2013; 37:658–65. 10.1002/gepi.21758 24114802 PMC4377079

[20] Birney E. Mendelian Randomization. Cold Spring Harb Perspect Med. 2022; 12:a041302. 10.1101/cshperspect.a041302 34872952 PMC9121891

[21] Yuan S, Larsson SC. Coffee and Caffeine Consumption and Risk of Kidney Stones: A Mendelian Randomization Study. Am J Kidney Dis. 2022; 79:9–14.e1. 10.1053/j.ajkd.2021.04.018 34690004

[22] Burgess S, Small DS, Thompson SG. A review of instrumental variable estimators for Mendelian randomization. Stat Methods Med Res. 2017; 26:2333–55. 10.1177/0962280215597579 26282889 PMC5642006

[23] Kintu C, Soremekun O, Kamiza AB, Kalungi A, Mayanja R, Kalyesubula R, Bagaya S B, Jjingo D, Fabian J, Gill D, Nyirenda M, Nitsch D, Chikowore T, Fatumo S. The causal effects of lipid traits on kidney function in Africans: bidirectional and multivariable Mendelian-randomization study. EBioMedicine. 2023; 90:104537. 10.1016/j.ebiom.2023.104537 37001235 PMC10070509

[24] Georgakis MK, Gill D. Mendelian Randomization Studies in Stroke: Exploration of Risk Factors and Drug Targets With Human Genetic Data. Stroke. 2021; 52:2992–3003. 10.1161/STROKEAHA.120.032617 34399585

[25] Zheng J, Zhang Y, Rasheed H, Walker V, Sugawara Y, Li J, Leng Y, Elsworth B, Wootton RE, Fang S, Yang Q, Burgess S, Haycock PC, et al. Trans-ethnic Mendelian-randomization study reveals causal relationships between cardiometabolic factors and chronic kidney disease. Int J Epidemiol. 2022; 50:1995–2010. 10.1093/ije/dyab203 34999880 PMC8743120

[26] Zhu GL, Xu C, Yang KB, Tang SQ, Tang LL, Chen L, Li WF, Mao YP, Ma J. Causal relationship between genetically predicted depression and cancer risk: a two-sample bi-directional mendelian randomization. BMC Cancer. 2022; 22:353. 10.1186/s12885-022-09457-9 35361153 PMC8973550

[27] Scosyrev E. Identification of causal effects using instrumental variables in randomized trials with stochastic compliance. Biom J. 2013; 55:97–113. 10.1002/bimj.201200104 23180483

[28] Boef AG, Dekkers OM, le Cessie S. Mendelian randomization studies: a review of the approaches used and the quality of reporting. Int J Epidemiol. 2015; 44:496–511. 10.1093/ije/dyv071 25953784

[29] Association analysis identifies 65 new breast cancer risk loci. Nature. 2017; 551:92–4. 10.1038/nature24284 29059683 PMC5798588

[30] Titova OE, Michaëlsson K, Vithayathil M, Mason AM, Kar S, Burgess S, Larsson SC. Sleep duration and risk of overall and 22 site-specific cancers: A Mendelian randomization study. Int J Cancer. 2021; 148:914–20. 10.1002/ijc.33286 32895918 PMC7821333

[31] Chen F, Wen W, Long J, Shu X, Yang Y, Shu XO, Zheng W. Mendelian randomization analyses of 23 known and suspected risk factors and biomarkers for breast cancer overall and by molecular subtypes. Int J Cancer. 2022; 151:372–80. 10.1002/ijc.34026 35403707 PMC9177773

[32] Ren Q, Luo F, Ge S, Chen P. Major depression disorder may causally associate with the increased breast cancer risk: Evidence from two-sample mendelian randomization analyses. Cancer Med. 2023; 12:1984–96. 10.1002/cam4.5043 35852181 PMC9883582

[33] Palmer TM, Lawlor DA, Harbord RM, Sheehan NA, Tobias JH, Timpson NJ, Davey Smith G, Sterne JA. Using multiple genetic variants as instrumental variables for modifiable risk factors. Stat Methods Med Res. 2012; 21:223–42. 10.1177/0962280210394459 21216802 PMC3917707

[34] Yuan K, Song W, Liu Z, Lin GN, Yu S. Mendelian Randomization and GWAS Meta Analysis Revealed the Risk-Increasing Effect of Schizophrenia on Cancers. Biology (Basel). 2022; 11:1345. 10.3390/biology11091345 36138824 PMC9495962

[35] Cai J, Li X, Wu S, Tian Y, Zhang Y, Wei Z, Jin Z, Li X, Chen X, Chen WX. Assessing the causal association between human blood metabolites and the risk of epilepsy. J Transl Med. 2022; 20:437. 10.1186/s12967-022-03648-5 36180952 PMC9524049

[36] Ren F, Shang Q, Zhao S, Yang C, Feng K, Liu J, Kang X, Zhang R, Wang X, Wang X. An exploration of the correlations between seven psychiatric disorders and the risks of breast cancer, breast benign tumors and breast inflammatory diseases: Mendelian randomization analyses. Front Psychiatry. 2023; 14:1179562. 10.3389/fpsyt.2023.1179562 37448488 PMC10338175

[37] Kelsey JL, Bernstein L. Epidemiology and prevention of breast cancer. Annu Rev Public Health. 1996; 17:47–67. 10.1146/annurev.pu.17.050196.000403 8724215

[38] Singletary KW, Gapstur SM. Alcohol and breast cancer: review of epidemiologic and experimental evidence and potential mechanisms. JAMA. 2001; 286:2143–51. 10.1001/jama.286.17.2143 11694156

[39] Yager JD, Davidson NE. Estrogen carcinogenesis in breast cancer. N Engl J Med. 2006; 354:270–82. 10.1056/NEJMra050776 16421368

[40] Boyd NF, Guo H, Martin LJ, Sun L, Stone J, Fishell E, Jong RA, Hislop G, Chiarelli A, Minkin S, Yaffe MJ. Mammographic density and the risk and detection of breast cancer. N Engl J Med. 2007; 356:227–36. 10.1056/NEJMoa062790 17229950

[41] Pierce BL, Burgess S. Efficient design for Mendelian randomization studies: subsample and 2-sample instrumental variable estimators. Am J Epidemiol. 2013; 178:1177–84. 10.1093/aje/kwt084 23863760 PMC3783091

[42] Bowden J, Davey Smith G, Burgess S. Mendelian randomization with invalid instruments: effect estimation and bias detection through Egger regression. Int J Epidemiol. 2015; 44:512–25. 10.1093/ije/dyv080 26050253 PMC4469799

[43] Higgins JP, Thompson SG, Deeks JJ, Altman DG. Measuring inconsistency in meta-analyses. BMJ. 2003; 327:557–60. 10.1136/bmj.327.7414.557 12958120 PMC192859

[44] Greco M FD, Minelli C, Sheehan NA, Thompson JR. Detecting pleiotropy in Mendelian randomisation studies with summary data and a continuous outcome. Stat Med. 2015; 34:2926–40. 10.1002/sim.6522 25950993

[45] Musicco M, Adorni F, Di Santo S, Prinelli F, Pettenati C, Caltagirone C, Palmer K, Russo A. Inverse occurrence of cancer and Alzheimer disease: a population-based incidence study. Neurology. 2013; 81:322–8. 10.1212/WNL.0b013e31829c5ec1 23843468

[46] Hanson HA, Horn KP, Rasmussen KM, Hoffman JM, Smith KR. Is Cancer Protective for Subsequent Alzheimer’s Disease Risk? Evidence From the Utah Population Database. J Gerontol B Psychol Sci Soc Sci. 2017; 72:1032–43. 10.1093/geronb/gbw040 27101831 PMC5926998

[47] Ganguli M. Cancer and Dementia: It’s Complicated. Alzheimer Dis Assoc Disord. 2015; 29:177–82. 10.1097/WAD.0000000000000086 25710132 PMC4437917

[48] Majd S, Power J, Majd Z. Alzheimer’s Disease and Cancer: When Two Monsters Cannot Be Together. Front Neurosci. 2019; 13:155. 10.3389/fnins.2019.00155 30881282 PMC6407038

[49] Zhang Y, Kong W, Jiang J. Prevention and treatment of cancer targeting chronic inflammation: research progress, potential agents, clinical studies and mechanisms. Sci China Life Sci. 2017; 60:601–16. 10.1007/s11427-017-9047-4 28639101

[50] Sanabria-Castro A, Alvarado-Echeverría I, Monge-Bonilla C. Molecular Pathogenesis of Alzheimer’s Disease: An Update. Ann Neurosci. 2017; 24:46–54. 10.1159/000464422 28588356 PMC5448443

[51] Sigal A, Rotter V. Oncogenic mutations of the p53 tumor suppressor: the demons of the guardian of the genome. Cancer Res. 2000; 60:6788–93. 11156366

[52] Blandino G, Di Agostino S. New therapeutic strategies to treat human cancers expressing mutant p53 proteins. J Exp Clin Cancer Res. 2018; 37:30. 10.1186/s13046-018-0705-7 29448954 PMC5815234

[53] Caricasole A, Bakker A, Copani A, Nicoletti F, Gaviraghi G, Terstappen GC. Two sides of the same coin: Wnt signaling in neurodegeneration and neuro-oncology. Biosci Rep. 2005; 25:309–27. 10.1007/s10540-005-2893-6 16307379

[54] García-Velázquez L, Arias C. The emerging role of Wnt signaling dysregulation in the understanding and modification of age-associated diseases. Ageing Res Rev. 2017; 37:135–45. 10.1016/j.arr.2017.06.001 28624530

[55] Marrie RA, Reider N, Cohen J, Stuve O, Trojano M, Sorensen PS, Reingold SC, Cutter G. A systematic review of the incidence and prevalence of cancer in multiple sclerosis. Mult Scler. 2015; 21:294–304. 10.1177/1352458514564489 25533302 PMC4429168

[56] Hajiebrahimi M, Montgomery S, Burkill S, Bahmanyar S. Risk of Premenopausal and Postmenopausal Breast Cancer among Multiple Sclerosis Patients. PLoS One. 2016; 11:e0165027. 10.1371/journal.pone.0165027 27776164 PMC5077134

[57] Marrie RA, Maxwell C, Mahar A, Ekuma O, McClintock C, Seitz D, Webber C, Groome PA. Cancer Incidence and Mortality Rates in Multiple Sclerosis: A Matched Cohort Study. Neurology. 2021; 96:e501–12. 10.1212/WNL.0000000000011219 33239364

[58] Grytten N, Myhr KM, Celius EG, Benjaminsen E, Kampman M, Midgard R, Vatne A, Aarseth JH, Riise T, Torkildsen Ø. Risk of cancer among multiple sclerosis patients, siblings, and population controls: A prospective cohort study. Mult Scler. 2020; 26:1569–80. 10.1177/1352458519877244 31573834

[59] Bosco-Lévy P, Foch C, Grelaud A, Sabidó M, Lacueille C, Jové J, Boutmy E, Blin P. Incidence and risk of cancer among multiple sclerosis patients: A matched population-based cohort study. Eur J Neurol. 2022; 29:1091–9. 10.1111/ene.15226 34936169

[60] Hawkes CH. Smoking is a risk factor for multiple sclerosis: a metanalysis. Mult Scler. 2007; 13:610–5. 10.1177/1352458506073501 17548439

[61] Marrie R, Horwitz R, Cutter G, Tyry T, Campagnolo D, Vollmer T. High frequency of adverse health behaviors in multiple sclerosis. Mult Scler. 2009; 15:105–13. 10.1177/1352458508096680 18845651

[62] Khan N, Afaq F, Mukhtar H. Lifestyle as risk factor for cancer: Evidence from human studies. Cancer Lett. 2010; 293:133–43. 10.1016/j.canlet.2009.12.013 20080335 PMC2991099

[63] Ragonese P, Aridon P, Vazzoler G, Mazzola MA, Lo Re V, Lo Re M, Realmuto S, Alessi S, D’Amelio M, Savettieri G, Salemi G. Association between multiple sclerosis, cancer risk, and immunosuppressant treatment: a cohort study. BMC Neurol. 2017; 17:155. 10.1186/s12883-017-0932-0 28789625 PMC5549380

[64] Hauser SL, Bar-Or A, Comi G, Giovannoni G, Hartung HP, Hemmer B, Lublin F, Montalban X, Rammohan KW, Selmaj K, Traboulsee A, Wolinsky JS, Arnold DL, et al, and OPERA I and OPERA II Clinical Investigators. Ocrelizumab versus Interferon Beta-1a in Relapsing Multiple Sclerosis. N Engl J Med. 2017; 376:221–34. 10.1056/NEJMoa1601277 28002679

[65] Montalban X, Hauser SL, Kappos L, Arnold DL, Bar-Or A, Comi G, de Seze J, Giovannoni G, Hartung HP, Hemmer B, Lublin F, Rammohan KW, Selmaj K, et al, and ORATORIO Clinical Investigators. Ocrelizumab versus Placebo in Primary Progressive Multiple Sclerosis. N Engl J Med. 2017; 376:209–20. 10.1056/NEJMoa1606468 28002688

[66] Leist TP, Comi G, Cree BA, Coyle PK, Freedman MS, Hartung HP, Vermersch P, Casset-Semanaz F, Scaramozza M, and oral cladribine for early MS (ORACLE MS) Study Group. Effect of oral cladribine on time to conversion to clinically definite multiple sclerosis in patients with a first demyelinating event (ORACLE MS): a phase 3 randomised trial. Lancet Neurol. 2014; 13:257–67. 10.1016/S1474-4422(14)70005-5 24502830

[67] Cohen JA, Coles AJ, Arnold DL, Confavreux C, Fox EJ, Hartung HP, Havrdova E, Selmaj KW, Weiner HL, Fisher E, Brinar VV, Giovannoni G, Stojanovic M, et al, and CARE-MS I investigators. Alemtuzumab versus interferon beta 1a as first-line treatment for patients with relapsing-remitting multiple sclerosis: a randomised controlled phase 3 trial. Lancet. 2012; 380:1819–28. 10.1016/S0140-6736(12)61769-3 23122652

[68] Fletcher JM, Lalor SJ, Sweeney CM, Tubridy N, Mills KH. T cells in multiple sclerosis and experimental autoimmune encephalomyelitis. Clin Exp Immunol. 2010; 162:1–11. 10.1111/j.1365-2249.2010.04143.x 20682002 PMC2990924

[69] Stephens LA, Malpass KH, Anderton SM. Curing CNS autoimmune disease with myelin-reactive Foxp3+ Treg. Eur J Immunol. 2009; 39:1108–17. 10.1002/eji.200839073 19350586

[70] Knochelmann HM, Dwyer CJ, Bailey SR, Amaya SM, Elston DM, Mazza-McCrann JM, Paulos CM. When worlds collide: Th17 and Treg cells in cancer and autoimmunity. Cell Mol Immunol. 2018; 15:458–69. 10.1038/s41423-018-0004-4 29563615 PMC6068176

[71] Senkevich K, Bandres-Ciga S, Yu E, Liyanage UE, Noyce AJ, Gan-Or Z, and International Parkinson Disease Genomics Consortium (IPDGC). No Evidence for a Causal Relationship Between Cancers and Parkinson’s Disease. J Parkinsons Dis. 2021; 11:801–9. 10.3233/JPD-202474 33646179 PMC9719261

[72] Wang T. The link between Parkinson’s disease and breast and prostate cancers: A meta-analysis. Int J Neurosci. 2015; 125:895–903. 10.3109/00207454.2014.986265 25387067

[73] Bajaj A, Driver JA, Schernhammer ES. Parkinson’s disease and cancer risk: a systematic review and meta-analysis. Cancer Causes Control. 2010; 21:697–707. 10.1007/s10552-009-9497-6 20054708

[74] Chen C, Zheng H, Hu Z. Association between Parkinson’s disease and risk of prostate cancer in different populations: An updated meta-analysis. Sci Rep. 2017; 7:13449. 10.1038/s41598-017-13834-x 29044216 PMC5647429

[75] Huang P, Yang XD, Chen SD, Xiao Q. The association between Parkinson’s disease and melanoma: a systematic review and meta-analysis. Transl Neurodegener. 2015; 4:21. 10.1186/s40035-015-0044-y 26535116 PMC4631109

[76] Dube U, Ibanez L, Budde JP, Benitez BA, Davis AA, Harari O, Iles MM, Law MH, Brown KM, Cruchaga C, 23andMe Research Team, and Melanoma-Meta-analysis Consortium. Overlapping genetic architecture between Parkinson disease and melanoma. Acta Neuropathol. 2020; 139:347–64. 10.1007/s00401-019-02110-z 31845298 PMC7379325

[77] Freedman DM, Wu J, Chen H, Engels EA, Enewold LR, Freedman ND, Goedert JJ, Kuncl RW, Gail MH, Pfeiffer RM. Associations between cancer and Parkinson’s disease in U.S. elderly adults. Int J Epidemiol. 2016; 45:741–51. 10.1093/ije/dyw016 26989123 PMC5841885

[78] Basta I, Pekmezovic T, Peric S, Nikolic A, Rakocevic-Stojanovic V, Stevic Z, Marjanovic I, Lavrnic D. Extrathymic malignancies in a defined cohort of patients with myasthenia gravis. J Neurol Sci. 2014; 346:80–4. 10.1016/j.jns.2014.07.060 25129207

