# Supplementary material for: An exploration of causal relationships between nine neurological diseases and the risk of breast cancer: a Mendelian randomization study
Source: Aging (Albany NY). 2024 Apr 24;16(8):7101–18. doi: 10.18632/aging.205745 (PMC11087125; doi:10.18632/aging.205745)
Supplement: Supplementary Figures [file aging-16-205745-s001.pdf]

SUPPLEMENTARY FIGURES

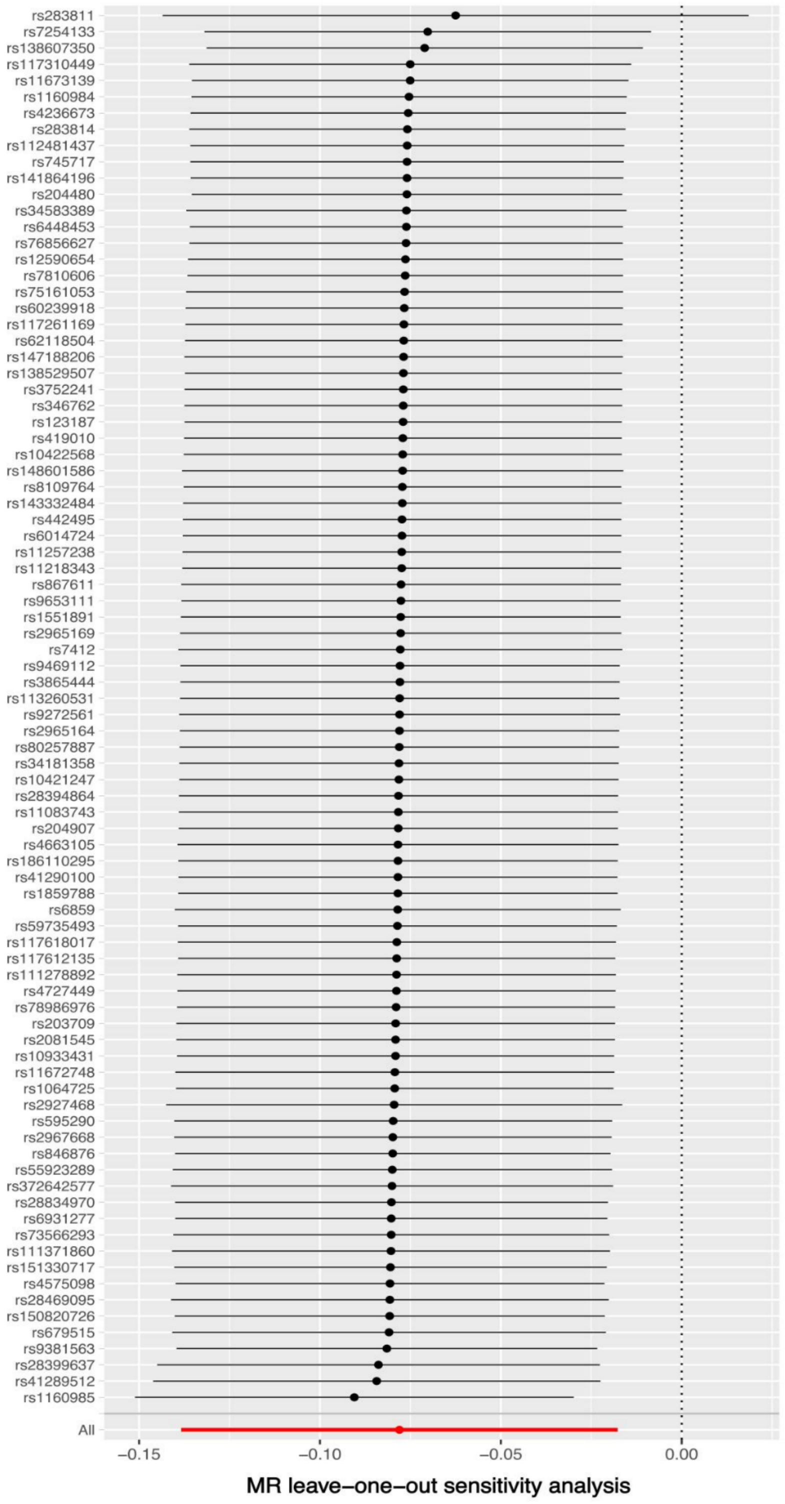

Supplementary Figure 1. Leave-one-out analysis of Alzheimer's disease and overall breast cancer risk.

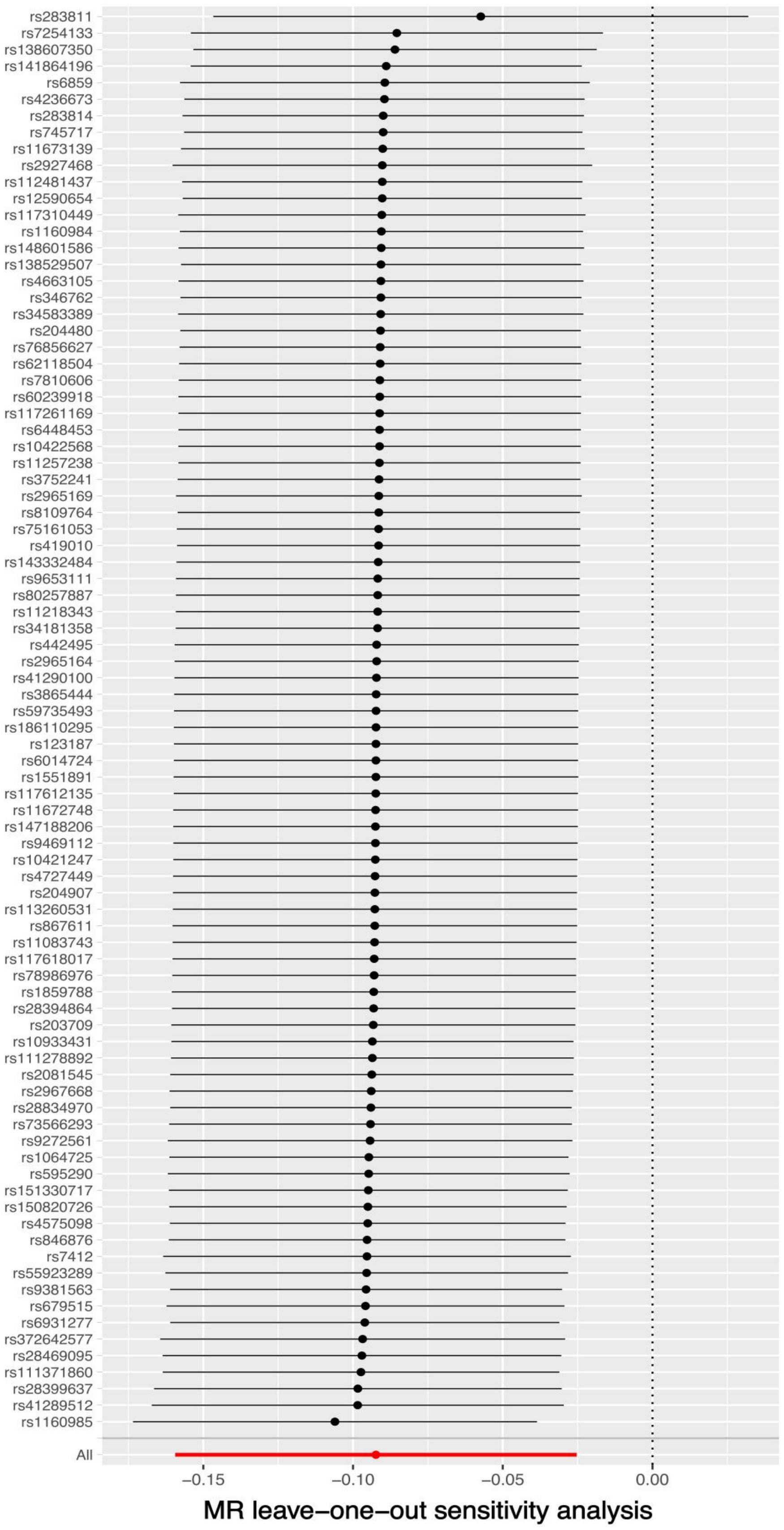

Supplementary Figure 2. Leave-one-out analysis of Alzheimer's disease and ER+ breast cancer risk.

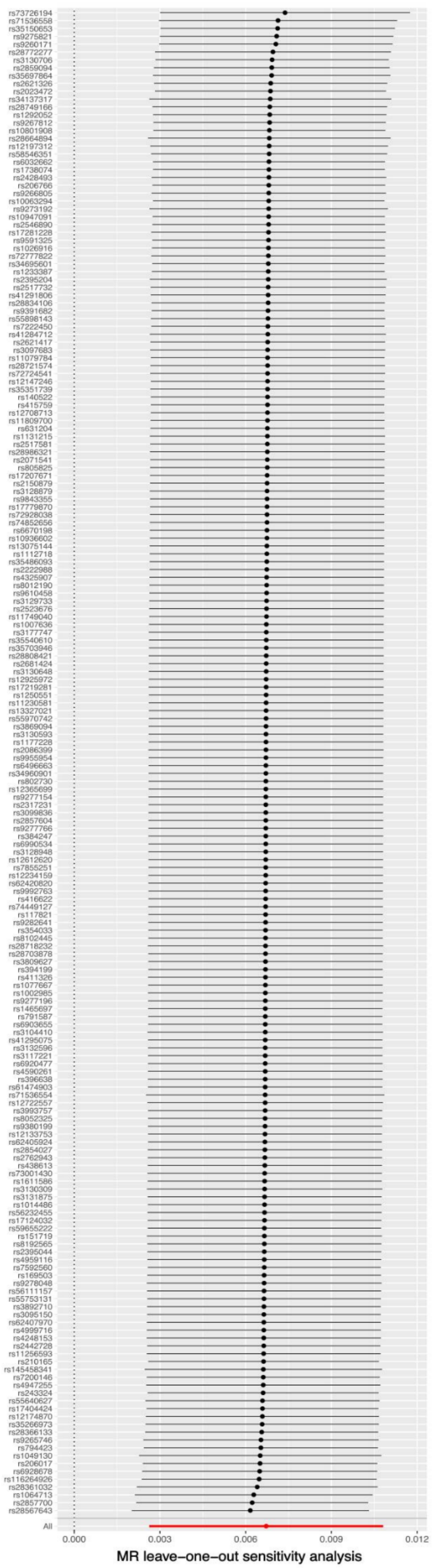

Supplementary Figure 3. Leave-one-out analysis of multiple sclerosis and ER+ breast cancer risk.
