# Supplementary material for: An exploration of causal relationships between nine neurological diseases and the risk of breast cancer: a Mendelian randomization study
Source: Aging (Albany NY). 2024 Apr 24;16(8):7101–18. doi: 10.18632/aging.205745 (PMC11087125; doi:10.18632/aging.205745)
Supplement: Supplementary Tables [file aging-16-205745-s002.pdf]

SUPPLEMENTARY TABLES

Supplementary Table 1. Detailed information on GWAS data for neurological disease.

Neurological disease	Year	Sample size	Consortium	Link
Alzheimer's disease	2019	455258	Complex Trait Genetics lab	https://pubmed.ncbi.nlm.nih.gov/30617256/
Multiple sclerosis	2019	115803	International Multiple Sclerosis Genetics Consortium	https://gwas.mrcieu.ac.uk/datasets/ieu-b-18/
Parkinson's disease	2019	482730	International Parkinson's Disease Genomics Consortium	https://gwas.mrcieu.ac.uk/datasets/ieu-b-7/
Myasthenia gravis	2021	217288	FinnGen consortium	https://gwas.mrcieu.ac.uk/datasets/finn-b-G6_MYASTHENIA/
Generalized epilepsy	2021	214313	FinnGen consortium	https://gwas.mrcieu.ac.uk/datasets/finn-b-GE/
Intracerebral haemorrhage	2021	202833	FinnGen consortium	https://gwas.mrcieu.ac.uk/datasets/finn-b-I9_ICH/
Cerebral atherosclerosis	2021	203172	FinnGen consortium	https://gwas.mrcieu.ac.uk/datasets/finn-b-I9_CERATHER/
Brain glioblastoma	2021	218792	FinnGen consortium	https://gwas.mrcieu.ac.uk/datasets/finn-b-C3_GBM/
Benign meningeal tumor	2021	218792	FinnGen consortium	https://gwas.mrcieu.ac.uk/datasets/finn-b-CD2_BENIGN_MENINGES_CEREBRAL/

Supplementary Table 2. Detailed information on GWAS data for breast cancer.

Outcome	Year	Sample size	Consortium	Link
Overall breast cancer	2017	106776	Breast Cancer Association Consortium	https://gwas.mrcieu.ac.uk/datasets/ieu-a-1129/
ER+ breast cancer	2017	83691	Breast Cancer Association Consortium	https://gwas.mrcieu.ac.uk/datasets/ieu-a-1132/
ER- breast cancer	2017	55149	Breast Cancer Association Consortium	https://gwas.mrcieu.ac.uk/datasets/ieu-a-1135/

Supplementary Table 3. The MR analyses of neurological diseases and ER+ breast cancer risk from MR Egger and weighted median methods.

Neurological diseases	Used SNPs	MR Egger		Weighted median		$P_{heterogeneity}$	$P_{pleiotropy}$
		OR(95% CI)	*P*-value	OR(95% CI)	*P*-value		
Alzheimer's disease	86	0.884(0.809-0.965)	0.007	0.875(0.802-0.954)	0.003	0.100	0.284
Multiple sclerosis	192	1.006(1.001-1.012)	0.019	1.005(0.999-1.011)	0.113	0.061	0.791
Parkinson's disease	25	1.046(0.942-1.162)	0.406	1.028(0.978-1.081)	0.273	0.006	0.525
Myasthenia gravis	8	0.991(0.968-1.015)	0.504	0.996(0.976-1.018)	0.742	0.199	0.190
Generalized epilepsy	12	1.010(0.978-1.043)	0.554	0.998(0.969-1.029)	0.908	0.718	0.097
Intracerebral haemorrhage	5	1.005(0.940-1.075)	0.891	1.031(0.977-1.087)	0.265	0.409	0.728
Cerebral atherosclerosis	7	1.005(0.995-1.015)	0.401	1.004(0.995-1.013)	0.358	0.379	0.755
Brain glioblastoma	8	0.993(0.975-1.012)	0.508	0.995(0.983-1.006)	0.351	0.078	0.659
Benign meningeal tumor	12	0.994(0.960-1.029)	0.742	1.008(0.980-1.038)	0.572	0.107	0.266

Supplementary Table 4. The MR analyses of neurological diseases and ER- breast cancer risk from MR Egger and weighted median methods.

Neurological diseases	Used SNPs	MR Egger		Weighted median		$P_{heterogeneity}$	$P_{pleiotropy}$
		OR(95% CI)	*P*-value	OR(95% CI)	*P*-value		
Alzheimer's disease	86	0.978(0.852-1.123)	0.756	0.981(0.854-1.126)	0.783	0.194	0.921
Multiple sclerosis	192	0.998(0.990-1.006)	0.608	0.996(0.987-1.006)	0.455	0.081	0.709
Parkinson's disease	26	1.013(0.890-1.153)	0.848	1.033(0.963-1.108)	0.361	0.285	0.939
Myasthenia gravis	8	1.020(0.980-1.062)	0.372	1.013(0.983-1.044)	0.406	0.212	0.558
Generalized epilepsy	12	1.029(0.961-1.101)	0.432	1.018(0.967-1.071)	0.499	0.089	0.858
Intracerebral haemorrhage	5	1.072(0.975-1.179)	0.245	1.065(0.982-1.154)	0.129	0.976	0.834
Cerebral atherosclerosis	7	1.006(0.991-1.020)	0.487	1.003(0.988-1.017)	0.714	0.796	0.561
Brain glioblastoma	8	0.992(0.971-1.014)	0.493	0.999(0.982-1.017)	0.936	0.569	0.330
Benign meningeal tumor	12	1.006(0.960-1.053)	0.817	1.010(0.966-1.056)	0.666	0.840	0.952
